# Use of Nonvascularized Autologous Fibular Strut Graft in the Treatment of Major Bone Defect after Periprosthetic Knee Fracture

**DOI:** 10.1155/2017/1650194

**Published:** 2017-05-18

**Authors:** Vincenzo Giordano, Bruno Parilha Coutinho, Mateus Kenji Miyahira, Felipe Serrão Mendes de Souza, Ney Pecegueiro do Amaral

**Affiliations:** ^1^Serviço de Ortopedia e Traumatologia Professor Nova Monteiro, Hospital Municipal Miguel Couto, Rio de Janeiro, RJ, Brazil; ^2^Núcleo Especializado de Ortopedia e Traumatologia, Clínica São Vicente, Rio de Janeiro, RJ, Brazil

## Abstract

We present the case of a patient who suffered a comminuted supracondylar periprosthetic femur fracture. The patient was an 86-year-old lady who suffered a minor fall at home and presented at our hospital with a right comminuted distal femur fracture around a total knee arthroplasty. The patient was submitted to a cruciate-sacrificing total knee replacement 6 years before at the same institution. Despite severe metaphyseal fragmentation and short distal fragment, the prosthesis was stable; thus, open fracture reduction and stabilization with internal fixation were performed. The surgical technique included the use of a nonvascularized autologous fibular strut graft as an augmentation technique in conjunction with double plating fixation. Clinically, patient presented a painless aligned knee 12 months after femur fixation, although she was not able to return to an independent level of activity. No pain involving the donor graft site was reported at the time of the most recent follow-up examination. This case study demonstrates the use of free nonvascularized autogenous fibular strut bone graft as an option to bridge major bone defects. This proved to be a relatively simple, not expensive procedure that can be done percutaneously and does not need high-quality training.

## 1. Introduction

The use of nonvascularized strut grafts to meet the challenging problem of bridging bone defects resulting from traumatic and nontraumatic conditions is not new [1, 2]. Among traumatic conditions, open fractures are more amenable to present bone defects, although some closed injuries can be complicated by the presence of bad bone stock and comminution, such as some periprosthetic fractures around the hip and knee.

Fractures of the distal femur after total knee arthroplasty (TKA) have been increasingly found [3, 4]. In general, stable total knee prosthesis should be preferably fixed by open reduction and internal fixation with a plate or an intramedullary (IM) nail [3, 5]. More recently, the use of anatomic distal femoral locking plates has been suggested as it permits early mobilization and allows for a better purchase of the implant [4]. Augmentation techniques, such as an IM cortical strut graft, a second medial plate, or both can be added mainly for mechanical purposes [3, 4, 6, 7].

In this paper, we present the case of a patient who suffered a comminuted supracondylar femur fracture 6 years after a primary TKA. The prosthesis was stable; thus, fracture reduction and stabilization with internal fixation were performed. The surgical technique is described in which a nonvascularized autologous fibular strut graft was used nontraditionally as an augmentation technique in conjunction with double plating fixation.

## 2. Case Report

An 86-year-old lady suffered a minor fall at home and was presented to our hospital with a right comminuted distal femur fracture around a TKA. The patient was submitted to a cruciate-sacrificing total knee replacement 6 years before at the same institution. After this surgery she was able to walk at home but not independently on the street. At the time of presentation, the patient was hemodynamically stable with a Glasgow Coma Score (GCS) of 15. On exam, she was noted to have Alzheimer's disease, diabetes mellitus, and some degree of cardiac dysfunction.

**Figure 1:**
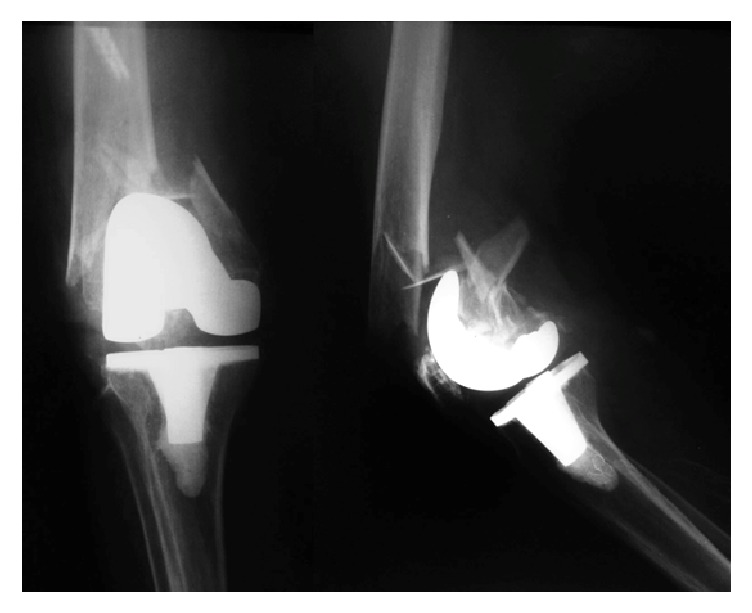
Presenting knee injury X-rays. Note the severe comminution of the metaphyseal area, including the medial wall of the distal femur. Radiologically, the femoral component seemed to be fixed.

Preoperative radiographs demonstrated severe fragmentation of the supracondylar metaphyseal bone with unacceptable medial wall comminution. The epiphyseal distal fragment was very short and presented the classic posterior displacement on the lateral view. There was no sign of loosening of the femoral component (Figure 1). Exams revealed the patient was an American Society of Anesthesiology grade III.

Patient was operated on under combined spinal epidural anesthesia and intravenous sedation. No tourniquet was used. The right fibula was harvested according to the technique described by Mukherjee et al. [8]. By using two separate incisions, 1 cm each at proximal and distal extent of proposed donor site, a segment of single fibula appropriate for the defect to be bridged was taken from a safe area of the bone without jeopardizing the associated neurovascular structures and proximal and distal tibiofibular joints. The free nonvascularized autogenous fibular strut bone graft measured about 21 cm. Before its use, the graft was divided unevenly into a smaller piece measuring about 9 cm and a larger piece measuring about 12 cm. Both ends of the larger piece of the graft were fashioned to fit at least 1 cm inside the medullary canal of the recipient bone. The smaller piece was left intact to be used as the medial metaphyseal distal femoral wall.

**Figure 2:**
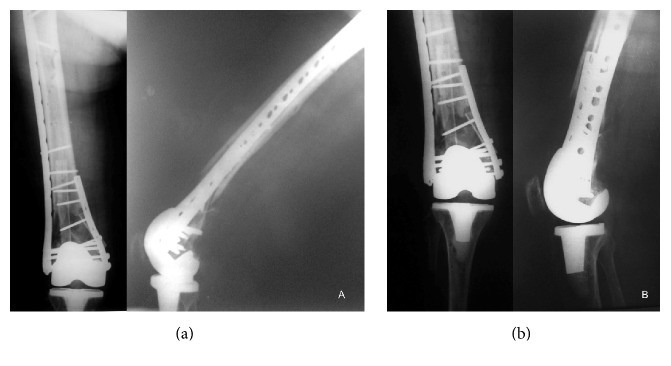
Immediate postoperative X-rays showing good reduction of the fracture, anatomic alignment of the articular surface, and rigid fixation. (a) Note the nonvascularized fibular strut graft. The larger piece was used to bridge the metaphyseal defect and the small piece to replace the medial wall of the distal femur. (b) The alignment of the knee joint was anatomically restored.

Following an anterolateral skin incision of 20 cm, a lateral parapatellar arthrotomy was performed and was continued proximally and distally up to the tibial tuberosity, according to the technique described by Krettek et al. [9]. The patella was medially retracted and the femur fracture was directly aligned by gentle manipulation. There were a metaphyseal bone defect about 3 cm and severe comminution of the medial wall of the distal femur. The femoral prostheses were confirmed to be well fixed. The medullary cavity of the proximal fragment was opened and the larger piece of the fibular strut graft was inserted into the medullary cavity proximally. Distally the fibular graft was positioned in the middle of the femoral condyles. The reduction was temporarily kept with smooth K-wires. The smaller piece of the fibular graft was positioned to replace the medial distal femoral wall and then fixed by a 10-hole small fragment dynamic compression plate (Baumer, Mogi Mirim, Brazil) holding it to the recipient bone. Finally the periprosthetic fracture was fixed with an anatomic locking plate (GMReis, Campinas, Brazil) applied on the lateral surface of the distal femur (Figure 2). No cancellous bone graft or bone substitute was used.

**Figure 3:**
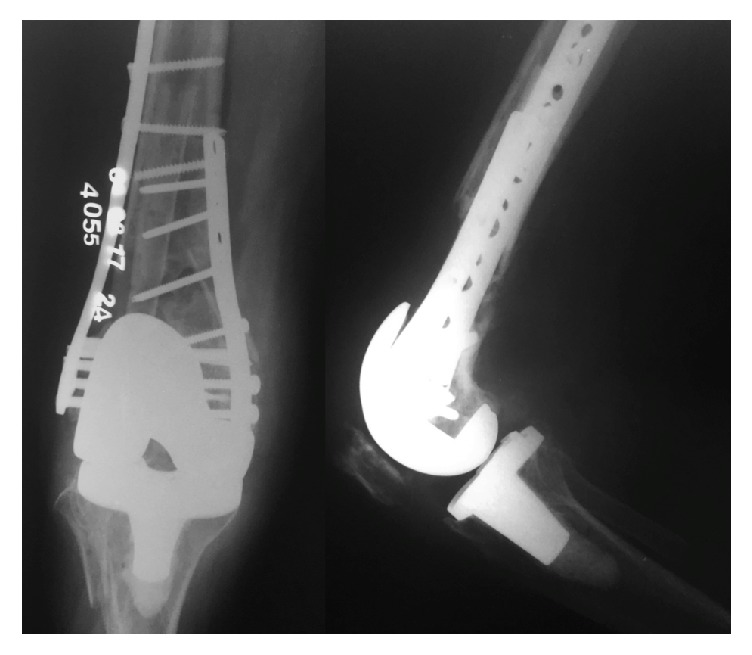
Final follow-up radiographs showing osseointegration of the fibular graft with definite bridging of the distal metaphyseal femur defect.

Postoperatively, patient started rehabilitation protocol and was discharged 72 hours after the procedure. Clinical and radiological controls were performed at 1, 3, 6, and 12 weeks and then at 3, 6, and 12 months. Last X-rays demonstrated complete healing of the fracture with osseointegration of the fibular strut graft (Figure 3).

**Figure 4:**
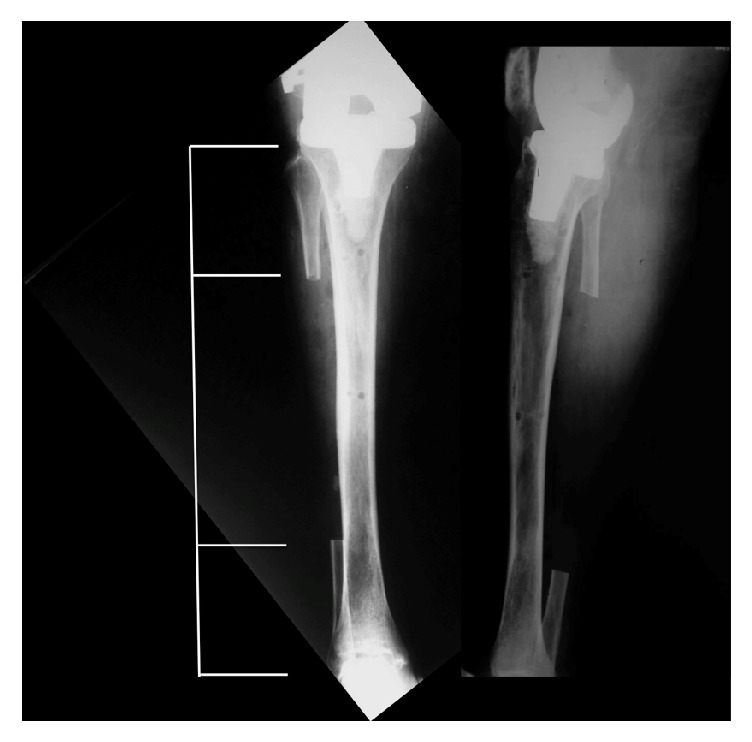
Patient had no pain involving the donor leg at the time of the most recent follow-up examination. Note the large resection of the fibular shaft.

Clinically patient presented a painless aligned knee, although she was not able to return to an independent level of activity. No pain involving the donor graft site was reported at the time of the most recent follow-up examination (Figure 4).

## 3. Discussion

Periprosthetic fractures around the knee are increasing in frequency as the number of primary knee replacements is continuously increasing [10]. The most common fracture after TKA occurs at the supracondylar area of the femur and can be complicated by osteoporosis, comminution, distal short fragment, and loose implant [3, 4, 10]. The ultimate goal of treating these injuries is fracture union with the preservation of a painless, stable, functional knee, without residual malalignment [4, 5]. Results are considered good if patients maintain at least a 90° range of motion with less than 2 cm of shortening, less than 5° of varus or valgus malalignment in the coronal plane, and less than 10° of malalignment in the sagittal plane [10].

Although some authors have reported on good results after nonoperative treatment, currently the only indication for this is in an elderly patient with a stable fracture pattern without displacement and a well-fixed component [10, 11]. The vast majority of distal femur periprosthetic fractures requires surgical intervention because of the high prevalence of progressive displacement, nonunion, and malalignment of the articular surface [11]. A wide variety of orthopedic devices may be used for fixation of these fractures. Conventional plates do not provide adequate stability due to the poor bone stock and are prone to high rates of fixation failure [4]. Retrograde-inserted IM nails may provide greater stability for the management of periprosthetic supracondylar femur fractures, especially in fracture patterns that contain a large medial fracture gap [5]. However, its use is restricted when the distal femur fracture occurs proximal to a posterior stabilized TKA component with a closed or narrow box. Recently, locking periarticular plates have become a popular treatment option because of having several advantages over the other options [4, 11]. In our opinion the major benefit of those implants is the effectiveness for stabilization of the distal fracture fragment independently of the type of prosthetic femoral component (if it is a closed or narrow box).

Thukral et al. described favorable clinical and radiological results in 27 of 31 periprosthetic supracondylar femoral fractures treated with DF-LCP (Distal Femur-Locking Compression Plate, Synthes Inc., Bettlach, Switzerland) [4]. However, it should be noted that while locking periarticular plates with multiple fixed-angle screws ensures a good option for the management of periprosthetic supracondylar femur fractures, there is a relatively high-risk of persistent instability, likely because of the limited bone stock for adequate distal fixation and the existence of osteoporosis and comminution at the metaphyseal area. In our patient there was a metaphyseal bone defect about 3 cm and severe comminution of the medial wall of the distal femur. Therefore, we decided to use a small part of the fibular graft to restore the medial wall and to add a medial plate over it to improve rigidity. We believe this could potentially reduce the risk of instability and associated complications, such as nonunion and malunion of the distal fragment. Ultimately, stable fixation and good local blood supply seem to be important cornerstones for early graft incorporation.

The large part of the nonvascularized fibular strut graft was used to bridge the metaphyseal defect. Like other authors, we fashioned the proximal end of the graft to fit inside the medullary canal of the recipient proximal fragment [4, 7]. The use of nonvascularized fibular strut grafts has been proven to be a reliable technique to reestablish bone continuity in segmental bone defects [1, 2, 4, 7, 12]. Many reconstruction procedures have been proposed to treat such conditions. The use of vascularized fibular graft is also a good option, with a small incidence of stress fracture. However, not all surgeons have the expertise, training, and facilities to perform this microsurgical procedure, which limits its use in many trauma situations. Other good options, like allogenic bone grafts and bone graft substitutes, also have limited use mainly due to its high cost [11, 13].

Complications when harvesting the fibular strut graft, such as common peroneal nerve damage, weakness of extensor hallucis longus, ankle instability, nonunion, and stress fracture have been reported [8, 12]. In order to prevent intraoperative problems during fibular harvest, it is important to preserve at least 5 to 6 cm of the proximal and the distal parts of the fibula [12, 14]. Retaining the distal fibula can prevent adverse effects on the distal tibiofibular syndesmosis and the ankle joint [15]. In addition, we feel that the biological approach for harvesting long free nonvascularized fibular graft as proposed by Mukherjee et al. reduces donor site morbidity and is safer than conventional approach [8].

The associated use of autograft has been proposed as an osteogenic stimulus with good results reported [4, 6, 7, 11, 13, 15]. Those authors describe the use of cancellous bone graft for augmentation of the fibular graft and speeding up the healing process. However, different from us all of them used fibular graft augmentation for large shaft defects of long bones, where bone healing is expected to be slow. It is known so far that the healing process in a shaft fracture faces the problem of recruiting cells to the area, from either the thin periosteum, surrounding muscle, endothelium, or blood [16]. In contrast, the damaged trabeculae in cancellous bone are surrounded by marrow, with readily available stromal cells that can differentiate to osteoblasts [16]. In our patient we decided to not use cancellous bone graft because the defect was basically metaphyseal and we did not anticipate any problems related to osseointegration of the fibular strut graft. Taraz-Jamshidi et al. achieved solid bone union in 15 patients with giant cell tumor of distal radius treated by en-block resection and reconstruction with nonvascularized fibular autograft without additional cancellous autograft [17].

## 4. Conclusion

We feel this is a simple, not expensive procedure, which can be used to bridge major bone defects. This proved to be a relatively simple technique that can be done percutaneously and does not need high-quality training.
